# Targeted Next-Generation Sequencing Combined With Circulating-Free DNA Deciphers Spatial Heterogeneity of Resected Multifocal Hepatocellular Carcinoma

**DOI:** 10.3389/fimmu.2021.673248

**Published:** 2021-06-15

**Authors:** Jianzhen Lin, Songhui Zhao, Dongxu Wang, Yang Song, Yue Che, Xu Yang, Jinzhu Mao, Fucun Xie, Junyu Long, Yi Bai, Xiaobo Yang, Lei Zhang, Jin Bian, Xin Lu, Xinting Sang, Jie Pan, Kai Wang, Haitao Zhao

**Affiliations:** ^1^ Department of Liver Surgery, State Key Laboratory of Complex Severe and Rare Disease, Peking Union Medical College Hospital, Chinese Academy of Medical Sciences and Peking Union Medical College, Beijing, China; ^2^ Pancreas Center, The First Affiliated Hospital of Nanjing Medical University, Pancreas Institute, Nanjing Medical University, Nanjing, China; ^3^ Department of Bioinformatics, OrigiMed, Shanghai, China; ^4^ Department of Radiology, Peking Union Medical College Hospital, Chinese Academy of Medical Sciences and Peking Union Medical College (CAMS & PUMC), Beijing, China

**Keywords:** hepatocellular carcinoma, heterogeneity, circulating-free DNA, somatic mutation, immunotherapy

## Abstract

**Background:**

Hepatocellular carcinoma (HCC) has a high risk of recurrence after surgical resection, particularly among patients with multifocal HCC. Genomic heterogeneity contributes to the early recurrence. Few studies focus on targeted next-generation sequencing (tNGS) to depict mutational footprints of heterogeneous multifocal HCC.

**Methods:**

We conducted tNGS with an ultra-deep depth on 31 spatially distinct regions from 11 resected multifocal HCC samples. Matched preoperative peripheral circulating-free DNA (cfDNA) were simultaneously collected. Genomic alterations were identified and compared to depict the heterogeneity of multifocal HCC.

**Results:**

Widespread intertumoral heterogeneity of driver mutations was observed in different subfoci of multifocal HCC. The identified somatic mutations were defined as truncal drivers or branchy drivers according to the phylogenetic reconstruction. *TP53* and *TERT* were the most commonly altered truncal drivers in multifocal HCC, while the most frequently mutated branchy driver was *TSC2*. HCC patients with a higher level of intertumoral heterogeneity, defined by the ratio of truncal drivers less than 50%, had a shorter RFS after surgical resection (HR=0.17, p=0.028). Genome profiling of cfDNA could effectively capture tumor-derived driver mutations, suggesting cfDNA was a non-invasive strategy to gain insights of genomic alterations in patients with resected multifocal HCC.

**Conclusions:**

Truncal mutations and the level of genomic heterogeneity could be identified by tNGS panel in patients with resected multifocal HCC. cfDNA could serve as a non-invasive and real-time auxiliary method to decipher the intertumoral heterogeneity and identify oncodrivers of multifocal HCC.

## Background

Hepatocellular carcinoma (HCC) ranks the first leading pathological types of primary liver cancer and the third leading cause of cancer-associated death worldwide (1). Intrahepatic tumor dissemination is the most common route of metastasis for advanced HCC, resulting in little chance to undertake radical resection for these patients. Over 50% of the HCC patients were reported to have multifocal lesions at their initial diagnosis (2), and prognoses varied among patients with resected multifocal HCC.

Previous studies have been well demonstrated that HCC is featured as a highly heterogenous malignance (3) through comprehensive multi-omics analyses including whole-genome sequencing (WGS) and transcriptome sequencing (4). Different tumoral lesions in HCC exhibited *de novo* carcinogenesis or tended to share a common primary tumor clone. Clinically, patients with multifocal HCCs tend to have early recurrences with a grim prognosis despite receiving aggressive therapeutic interventions (5). Therefore, it is imperative to decipher the evolutionary relationship among multiple tumors of multifocal HCC based on molecular profiling, so that precise personalized therapy against multifocal HCC may be established (6, 7). Existing researches commonly utilized WGS or whole-exome sequencing (WES) to differentiate intratumor heterogeneity through clonal evolution analysis for genomic alterations. However, due to the high cost, it is currently difficult to be widely applied in clinical practice. In recent years, targeted next-generation sequencing (tNGS)-based panel, which captured critical cancer-related genes and structure variations, has been specifically designed and implemented in routine clinical practice (8). Nonetheless, this strategy has not been fully investigated in multifocal HCC. It is encouraging to apply tNGS panel to identify spatial heterogeneity and clonal relationship of multifocal HCC.

Herein, we applied an ultra-deep tNGS-based assay of Cancer Sequencing YS (CSYS) panel (9) to detect genomic alterations in 31 surgically resected tumor tissues and the paired preoperative circulating-free tumor DNA (cfDNA) samples from 11 multifocal HCC patients. We explored the mutational similarity and spatial heterogeneity on the basis of alterations of 466 cancer-related genes captured by CSYS panel. Finally, we further tracked clonal relationship from cfDNA and deciphered the clonal relationship among various tumor foci in these multifocal HCC patients.

## Materials and Methods

### Patients and Sample Collection

31 HCC tumor samples and 11 preoperative blood samples were obtained from 11 patients with resectable multifocal HCC who underwent primary and curative (tumor-free margin) surgical resections in our center (Peking Union Medical College Hospital, PUMCH). All tumor tissues were assessed by professional pathologists to confirm the diagnosis of HCC. The study’s protocol was approved by the Ethics Committee of PUMCH. All patients signed informed consent forms, and their clinical follow-up data were available.

### DNA Extraction and Sequencing

Only samples with estimated tumor purity >20% based on histopathological assessment were further subjected to genomic profiling. DNA was extracted from the fresh-frozen tumors and circulating leucocytes using a DNA Extraction Kit (Qiagen) according to the manufacturer’s protocols. Preoperative peripheral blood lymphocytes and plasma were separated through centrifugation at 1,600 g for 10 min. Supernatant plasma was then transferred to a new 2 mL centrifuge tube and centrifuged at 16,000 g for 10 min. MagMAX™ Cell-Free DNA isolation kit (Life Technologies, California, USA) was used to extract cfDNA in the plasma according to its manufacturer’s instructions. Tiangen whole blood DNA kit (Tiangen, Beijing, China) was used to extract DNA from peripheral blood lymphocytes according to the manufacturer’s instructions. DNA concentration was measured using Qubit dsDNA HS Assay kit or Qubit dsDNA BR Assay kit (Life Technologies, California, USA).

The tNGS panel (CSYS) for hybrid selection and the target-specific enrichment chip were designed and manufactured (OrigiMed, Shanghai) by custom pipeline. CSYS panel captured all coding exons of 466 key cancer-related genes and selected introns of 36 genes commonly rearranged in solid tumors (**Supplementary Table S1**). In addition, the probe density was increased to ensure high efficiency of capture in the conservatively low read depth region. With the input DNA at least 100ng for each library, CSYS panel was sequenced with a pre-set mean coverage of 900X for tumor DNA samples and 300X for matched blood samples on an Illumina NextSeq-500 Platform (Illumina Incorporated, San Diego, CA).

### Somatic Variants Calling and Tumor Mutation Burden (TMB)

Data quality was inspected and controlled by examining sequencing coverage and uniformity, and a suite of customized bioinformatics pipelines was applied as previous reports (10) for somatic variants calling, including single nucleotide variations (SNVs), short and long insertions/deletions (indels), copy number variations (CNVs) and gene rearrangements. We used MuTect (11) (version 1.7) to identify SNVs and used Pindel (12) (version 0.2.5) to identified indels. The lengths of short indels were required <50 bp, while those >50 bp were considered as long indels. For each alteration, we performed a manual review process to ensure no false positives or mistakes based on our in-house database. The annotations for these alterations were based on SnpEff 3.0 (13). CNVs were identified by Control-FREEC (14) (version 9.7) with the following parameters: step = 10,000 and Window = 50,000.

The processing for raw reads from cfDNA sequencing was followed as previously described (15). Briefly, cutadapt (version 1.18) (16) was used to filter out high-quality reads, and BWA (17) was used to map these reads into human genome by the reference from UCSC hg19 sequences. BaseRecalibrator tool from GATK (version 3.8) was applied to recalibrate base quality, and Picard was employed to remove PCR duplicates. Mutect2 was used to detect variants from cfDNA, and CNVs information was computed by CNVKit (18). All somatic variants were annotated by ANNOVAR (version 2017.07.17) with RefSeq (19).

TMB was estimated following the methods of Chalmers et al. (20). Briefly, SNVs and indels occurred in somatic and coding regions were counted. In order to reduce sampling noise, synonymous mutations were counted, while non-coding alterations and known germline alterations in dbSNP were excluded. To calculate the TMB per megabase (Mut/Mb), the total number of mutations counted was divided by the size of the coding region of the targeted territory.

### Determination of Potential Driver Mutation Genes

The definition of potential HCC-driver mutation genes referred to a published study. Briefly, potential driver mutation genes included significantly mutated in TCGA-LIHC (21) program of HCC’s genome (q<0.1), and mutations presented in TARGET database (v3.0, https://software.broadinstitute.org/cancer/cga/target).

### Clonality Analysis

To gain insights into the genetic phylogeny of multifocal HCC, tumor phylogeneties were reconstructed for each multifocal HCC case using LICHeE (Lineage Inference for Cancer Heterogeneity and Evolution) algorithm (22). LICHeE is a computational method to decipher cancer cell lineages using somatic mutations from tumor samples. The parameters of *LICHeE* were set as follows: minVAFPresent of 0.01, maxVAFAbsent of 0.01, and maximum number of trees of 1, and the others with default values. The phylogenetic tree was constructed according to the output tree of *LICHeE*, and length of trunks and branches were proportional to the corresponded mutations.

### Statistical Analysis

Assessments of differences in the means or medians of continuous variables were performed using SPSS software version 23 (IBM Corporation, Chicago, IL). The Mann-Whitney U test was employed to assess differences in the distributions of continuous variables between two groups. Fisher’s exact test was applied to examine the dependency of two binary variables. Spearman correlation tests were applied to analyze the relationship between two variables. A two-tailed p value <0.05 was considered significant. The “survival” R package was used for the survival analysis using the Kaplan-Meier estimator, and p values were calculated with the log-rank test. Estimations for hazard ratios (HRs) were applied with Kaplan-Meier estimator. The statistical analyses were performed using R software (R-3.5.1).

## Results

### Spatial Intertumoral Heterogeneity in Cancer Genome of Multifocal HCC

Surgically resected fresh-frozen tissues from 31 tumoral focal of 11 patients with pathologically-confirmed multifocal HCC (**Table 1**) were obtained to examine the genomic profiles. Preoperative peripheral blood leukocyte DNA was used as germline control for each patient. 9 of 11 patients had hepatitis B virus (HBV) infection, one patient had a history of hepatitis C virus (HCV) infection, and 3 patients were AFP-negative HCC at their initial diagnosis.

**Table 1:** Summary of baseline clinicopathological characteristics of 31 tumoral focal from 11 HCC patients.

Patient ID	Tumor ID	Age, yrs	Sex	Pathological differentiation	Vascular tumor thrombus	Hepatitis history	Hepatic cirrhosis	Preoperative AFP
HCC01	T1/T2	59	M	Moderate	None	None	None	571
HCC02	T1/T2	56	F	Moderate- poor	None	HBV	Yes	15114
HCC03	T1/T2/T3	64	M	Moderate	None	HBV	Yes	53.3
HCC04	T1/T2/T3	58	F	Poor	None	HBV	Yes	7.6
HCC05	T1/T2/T3	75	M	Well	None	HBV	Yes	184.8
HCC06	T1/T2/T3	61	M	Moderate	None	HCV	None	4.1
HCC07	T1/T2/T3	36	M	Moderate- poor	Portal vein	HBV	None	525.7
HCC08	T1/T2/T3	51	M	Moderate	Microvascular	HBV	Yes	1420
HCC09	T1/T2/T3	40	M	Poor	Portal vein	HBV	Yes	10.2
HCC010	T1/T2/T3	40	M	Moderate	Microvascular	HBV	Yes	663.6
HCC011	T1/T2/T3	55	M	Well- moderate	None	HBV	Yes	112.1

M, Male; F, Female; HBV, hepatitis B virus; HCV, hepatitis C virus; AFP, alpha-fetoprotein (ng/ml, range of normal values: 0-20).

**Figure 1:**
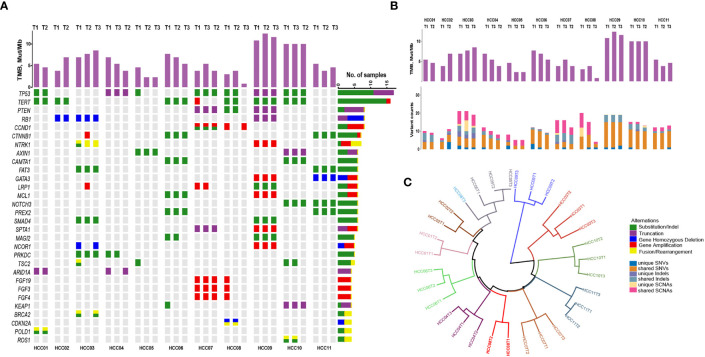
The mutational landscape and spatial heterogeneity of genetic divergence in different tumoral focal from 11 patients with resected multifocal HCC. **(A)** Concordance among somatic alterations (SNVs, indels and SCNAs) in detected cancer-related genes through tNGS panel among different primary intrahepatic tumor lesions. Stacked bar plots illustrate the tumor mutation burden (TMB) value of each tumor. **(B)** The absolute counts of somatic mutations (SNVs, indels and SCNAs) that are shared or subfoci-unique in all multifocal HCC samples. **(C)** Hierarchical clustering of all HCC samples based on the mutational landscape. T3 of HCC08 is separated away from other foci in patient.

CSYS was performed in these tumor and blood samples, with an average of 1304× sequencing depth. Among the coding exons of 466 genes and various genomic regions previously shown to be involved in HCC including *TERT* promoter, oncogenic fusions and hepatitis B or C virus genomic integrations, we identified a total of 132 somatic mutations across 98 genes (**Supplementary Table S2**), including 101 single-nucleotide variants (SNVs) and 31 short insertions and deletions (indels). High concordance among putative driver genes was observed among different foci in the individual patient (**Figure 1A**). As the most commonly altered driver genes, mutations in *TP53* and *TERT* were both identified in 7 of 11 (64%) HCC patients, and different tumor lesions shared *TP53* or *TERT* promoter alterations except for three patients (HCC05, HCC07 and HCC08’s T3). Besides, somatic copy number alterations (SCNAs) in these cancer-related genes were shared by different tumor foci in the same patient, especially in HCC07 and HCC09. Overall, we observed a high proportion of shared events in SNVs, indel, or SCNAs (**Figure 1B**), whereas three patients including HCC02, HCC03 and HCC09 had moderate numbers of unique somatic alterations. Moreover, we also estimated tumor mutation burden (TMB) for each tumor sample, and the average value of TMB was 5.4 (IQR: 3.8 – 7.7, SD: 2.9) Mut/Mb. Among this group of patients, the change of TMB is consistent with the mutation changes. For instance, the difference of mutation type between P02 and P09 is obvious, and the corresponding TMB change is also relatively significant (**Figure 1B**). These data implied that the genomic heterogeneity of multifocal HCC affected the assessment of TMB, which is a challenge for existing TMB-guided immune checkpoint blockade.

Hierarchical clustering of all HCC samples based on the genomic alterations revealed that almost all multifocal HCC patients in the present study could be categorized into intrahepatic metastasis spreading tumors, with the exception of T3 from HCC08 patient, which was considered as a multicenter originated tumor lesion (**Figure 1C**). These outcomes indicate that multifocal HCC shared large proportion of onco-driver mutations among different tumor lesions.

### Phylogenetic Reconstructions Identified Truncal and Branchy Drivers

**Figure 2:**
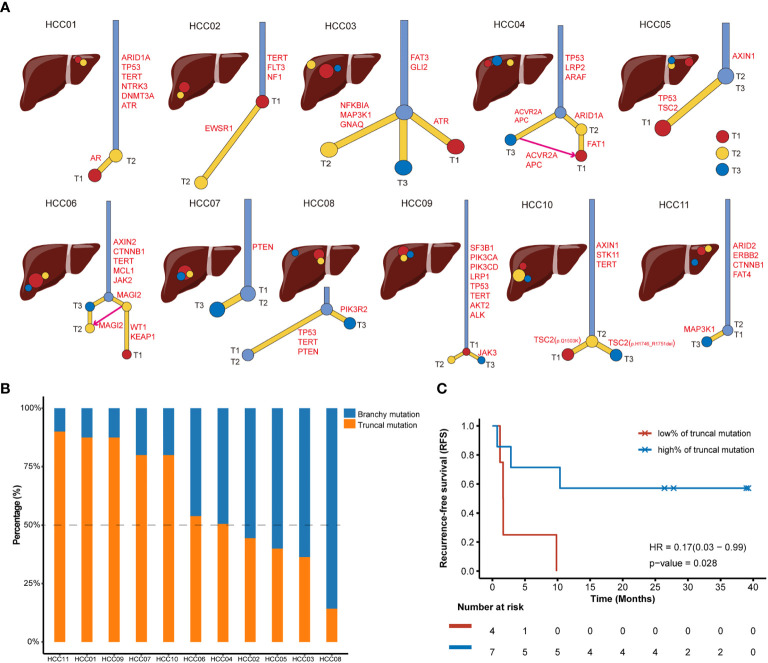
Phylogenetic trees of multifocal HCC cases and recurrence outcomes after radical surgical resections. **(A)** Phylogenetic trees are constructed using somatic mutations. Intrahepatic tumoral focal are arrayed in the liver around the anatomical diagram. T1 is the red circle, T2 is the yellow circle and T3 is the blue circle. The putative HCC driver mutations are also annotated in the trunks or branches of phylogenetic trees. **(B)** The tumor mutation burden (TMB) value and percentage of truncal mutations and branchy mutations in each patient with multifocal HCC. Blue bar and orange bar represent the proportion of branchy mutation and truncal mutation, respectively. **(C)** Kaplan-Meier curve showing poor recurrence-free survival (RFS) in patients with a low ratio (<50%) of truncal mutation (Log-rank test).

We dug into the substantial tumor heterogeneity and branched evolution in all 11 multifocal HCC patients to construct phylogenetic trees for these tumors. All HCC tumors showed a branched evolutionary pattern among the detected cancer-related genes (**Figure 2A**), which was consistent with previous studies proposed that genomic evolution of multifocal HCC was not a linear model (22). It should be emphasized that some focal tumors presented as an inconspicuous branching relationship under the narrow spectrum of cancer-related genes enrolled in CSYS, including HCC03, HCC05 and HCC08. In these patients, the inconspicuous branching relationship is mainly characterized by the short trunk of the evolutionary tree, indicating that there are fewer common mutations among the different lesions. Two patients (HCC04 and HCC06) showed a convergent tumor from other foci, because the T1 of these two patients was supposed to share the mutational features from both the other two superior tumors.

Next, putative driver mutations associated with HCC were mapped onto the phylogenetic trees to address whether specific driver genes were predominantly altered on trunks or branches. The identified somatic mutations could be classified into HCC drivers if it belonged to the frequently mutated genes of HCC that were proposed by *TCGA-LIHC* program (21) or presented in TARGET database (v3). To further investigate the evolutional phenotype of each driver mutation in individuals, we defined truncal drivers as mutations shared by all foci, which presented in trunks of the phylogenetic trees; and branchy drivers as mutations not shared by all foci or held by only one lesion. Therefore, both truncal drivers and branchy drivers were highlighted in each phylogenetic tree (**Figure 2A**). Among these 11 multifocal HCC patients, the most frequent truncal drivers were *TP53* mutants (4/11, 36.4%) and *TERT* promoter mutations (4/11, 36.4%), suggesting these two alterations occurs early in carcinogenesis of HCC. Moreover, *TSC2* mutations (3/11, 27.3%) was the most frequently mutated branchy driver, while sporadically mutated branchy drivers, such as *CCND1*, *CDKN2A*, *LRP1*, *MAP3K1* and *PTEN*, were also observed in multifocal HCC, which were supposed to drive the complicated heterogeneity and multiple phenotypes in HCC. Intriguingly, T1 and T3 of HCC10 showed distinct branches for these two tumor regions possessed different mutational loci in *TSC2* (**Figure 2A**), even though they shared a common ancestor in mutations including *AXIN1*, *STK11* and *TERT*.

To further quantify and appreciate the heterogeneity among different foci, we further determined the percentage of truncal drivers in all identified HCC drivers for each patient (**Figure 2B**). We supposed that the lower the proportion of truncal drivers were, the greater the genomic heterogeneities existed among different tumor lesions. The proportion of truncal drivers varied from patient to patient. Intriguingly, we observed a significantly poorer recurrence-free survival (RFS) after radical resection in patients with a low rate (<50%) of truncal drivers (HR=0.17, p=0.028, **Figure 2C**), suggesting highly heterogeneous multifocal HCC patients were speculated to have an underprivileged prognosis after receiving surgical resections. This outcome was consistent with clinical observations that patients with lower intratumor heterogeneity had better survival prognosis than those with higher level of heterogeneity (23), demonstrating that it’s imperative to timely infer the heterogeneous levels for resected multifocal HCC patients, and personalized postoperative adjuvant therapy should be considered for those with high risk of tumor recurrence determined by multi-regions genomic sequencing.

### cfDNA Tracked Mutations in Primary Multi-Focal HCC

Tumor truncal or branchy drivers may inform prognosis and recurrence risk after the surgical resection, which is worthwhile to be monitored for patients with resectable multifocal HCC. cfDNA has been proved as a non-invasive liquid biopsy for HCC’s somatic alterations (24). Herein, we simultaneously performed cfDNA sequencing at prior-surgery status on all 11 patients with available HCC foci tissues. On the day before the surgery, cfDNA detected a total of 46 SNVs and 5 indels, through an ultra-deep sequencing under an average depth of 5397×.

**Figure 3:**
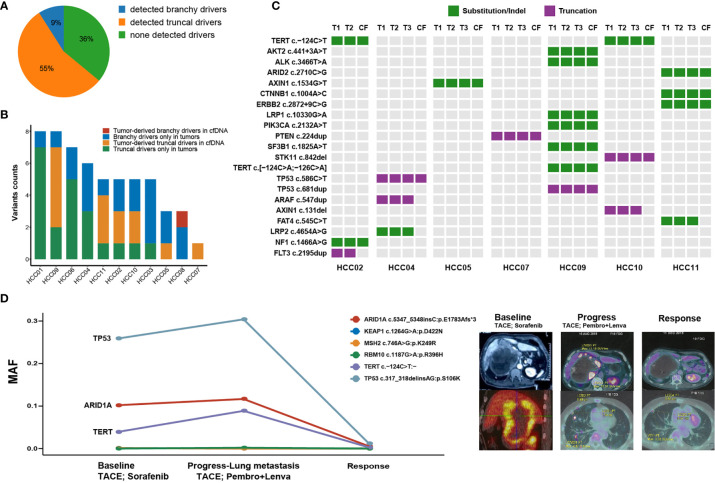
Overview of altered putative HCC driver mutations uncovered through circulating-free DNA (cfDNA). **(A)** The proportion of cfDNA-capturing tumor-derived driver mutations in 11 multifocal HCC patients. **(B)** The absolute counts of somatic HCC driver mutations that are simultaneously or independently captured by tumor tissues and cfDNA. **(C)** The distribution of HCC driver mutations that were mutant in at least one foci of each patient which partly presented in circulating-free (CF) samples. **(D)** Longitudinal tracking of tumor progression and therapeutic response by cfDNA to pembrolizumab plus lenvatinib in one patient with metastatic HCC. The line chart shows the mutational allele frequencies (MAFs) of major driver mutations in cfDNA which occurred at the time of baseline, disease progression with emerging lung metastasis, while almost absented at the time of objective response.

We focused on tumor-derived driver mutations captured by cfDNA in each patient. 7 of 11 (64%) multifocal HCC patients had cfDNA captured tumor-derived driver mutations, while branchy drivers were detected in only one patient (HCC08). This could be perfectly explained by previous analyses of genomic of multifocal tumors, whose results indicated that it was MO-HCC without truncal drivers (**Figure 3A**). For 6 patients with cfDNA-detectable truncal drivers, ubiquitous variants were commonly observed than unique variants that only existed in one or some of the tumors (**Figure 3B**). Importantly, cfDNA-captured mutational loci were highly consistent with these alterations occurred in tumor tissues (**Figure 3C**), suggesting that cfDNA could possibly retrieve intertumoral genomic heterogeneities and might be utilized to capture truncal drivers in tumor specimens of multifocal HCC.

Finally, we explored whether cfDNA could be used for discovering or tracking potential driver variants by dynamic monitors during antitumor treatment of HCC patients. Herein, we dynamically tracked tumor mutations in 3 cfDNA samples at different time-points from one advanced HCC patient who achieved the objective response after receiving combinational treatment of lenvatinib plus pembrolizumab (**Figure 3D**). The patient’s tumor sample is a large lesion that includes multiple regions with different densities under CT. At baseline of the initial cfDNA, this patient received one dose of transhepatic arterial chemotherapy and embolization (TACE) and then was treated with sorafenib for two months. His disease progressed with newly emerging metastasis in lungs. The second cfDNA analysis found mutation allele frequencies (MAFs) of three ubiquitous somatic variations (*TP53*, *ARID1A* and *TERT*) elevated in the circulating blood when compared with the initial cfDNA. Then, he received the second TACE treatment and was simultaneously treated by pembrolizumab (200 mg/3 weeks) combined with lenvatinib (8 mg/day) for the next two months. Along with the responsive status of obviously shrunk tumors, the third cfDNA analysis found extremely low MAFs of all previously detected ubiquitous somatic variations. Therefore, cfDNA preliminarily provided a promising tool to dynamically track genomic alterations or even truncal driving mutations during immunotherapy for HCC patients, and thereby, could inform disease progression or therapeutic responses.

## Discussion

This study investigated intertumoral genomic heterogeneities based on ultra-deep tNGS captured 466 cancer-related genes in patients with resected multifocal HCC. We deciphered an evolutionary trajectory in multifocal HCC through this methodology. We found varying levels of intertumoral genomic heterogeneity existed in different foci of multifocal HCC, even though only putative cancer-driving genes were considered. Of importance, these results indicate that diverse drivers in multifocal HCC are the underlying contributors that lead to the heterogeneity of clinical prognosis and therapeutic efficacy.

Our study proposed a clinical application model and analysis method to distinguish truncal or branchy drivers among different subfoci of multifocal HCC. Frequently mutated somatic driver mutations, including *TP53* and *TERT*, play a core role in carcinogenesis and tumor progression in HCC, which are still indestructible drug targets for HCC treatment. Besides, *TSC1* or *TSC2* alterations seems to be heterogeneity makers to hatch out subclones of primary HCC lesions. HCC patients carrying *TSC1/2* mutations were demonstrated to predispose a deregulated mTOR activity, and thereby inhibition of mTOR signaling has been widely investigated in clinical trials (25, 26). However, mTOR inhibitor like everolimus showed an unsatisfactory efficacy in advanced HCC patients (27), possibly due to its brachy role in HCC’s progression. As such, the data strongly supported that comprehensive insights of genetic landscape for multifocal HCC could reveal the most crucial target to facilitate the design of combinational targeted therapies (28, 29).

To explore the varying survival prognosis of resected multifocal HCC, we demonstrated the proportion of truncal drivers could be used as an assessable assay for intertumoral genomic heterogeneity through tNGS, and low ratio of truncal drivers (<50%) informed a significantly elevated recurrence risk after surgical resections for multifocal HCC patients. Accumulating evidences have revealed that intertumoral heterogeneity in multifocal HCC would foster tumor evolution, metastasis and chemotherapeutic resistance, suggesting that molecular heterogeneity is a key challenge in HCC treatment (30, 31). Considering the increased cost-effectiveness of tNGS, due to it is a lower cost, shorter cycle time and higher operability than either WES or WGS, this strategy provides a solution for the clinical application and personalized postoperative managements for patients with resected multifocal HCC.

In addition, preoperative cfDNA-based detection could sensitively capture most of the tumor-derived driver mutations, suggesting ultra-depth cfDNA could serve as a non-invasive and real-time auxiliary method to decipher the intertumoral heterogeneity and identify oncodrivers of multifocal HCC. It should be noted that in view of the limited capture of ctDNA, not all cancer species can effectively identify tumor heterogeneity. Whether circulating tumor DNA (ctDNA) profile could represent these ITH in different tumor type still needs to be confirmed by further research (32). Besides, we used cfDNA to dynamically monitor the therapeutic effect in a case with unresectable and metastatic HCC, implying that it is promising to track patients’ therapeutic responses through cfDNA detection. Although cfDNA demonstrated lower mutation detection efficiency, less genetically informative and robust repeatability than tumor tissues biopsy, cfDNA could provide a more comprehensive mutational footprints by revealing intertumoral heterogeneity in multifocal HCC (24). Impressively, cfDNA levels fluctuated consistently with pathophysiological conditions (33), bringing an emerging path to meet the demand of alpha-fetoprotein negative HCC patients.

## Conclusion

In conclusion, multifocal HCC shows a significant intertumoral genomic heterogeneity among tumor-associated genes and driver mutations. Through performing ultra-deep tNGS on global foci, both truncal and branchy drivers can be economically and effectively identified, which potentially provide a basis for decision-making for personalized therapy at postoperative or recurrent stage.

## Data Availability Statement

The datasets presented in this study can be found in online repositories. The names of the repository/repositories and accession number(s) can be found in the article/**Supplementary Material**.

## Ethics Statement 

The studies involving human participants were reviewed and approved by Peking union medical college hospital. The patients/participants provided their written informed consent to participate in this study. Written informed consent was obtained from the individual(s) for the publication of any potentially identifiable images or data included in this article.

## Author Contributions

JZL, SHZ and DXW collected the data and wrote the manuscript. YS, YC, XY, JZM, FCX, JYL, XBY helped to collect literature and participated in discussions. XBY, LZ, JB, XL, XTS, KW, JP and HTZ designed and verified the study. KW, JP and HTZ finally examined the study. All authors contributed to the article and approved the submitted version.

## Funding

HZ is supported by the International Science and Technology Cooperation Projects (2016YFE0107100 and 2015DFA30650), the CAMS Innovation Fund for Medical Science (CIFMS) (2017-I2M-4-003), the Beijing Natural Science Foundation (L172055), the National Ten-thousand Talent Program, the Beijing Science and Technology Cooperation Special Award Subsidy Project and the CAMS Initiative for Innovative Medicine (CAMS-2018-I2M-3-001).

## Conflict of Interest

SZ, YC and KW were employed by OrigiMed, Shanghai, China.

The remaining authors declare that the research was conducted in the absence of any commercial or financial relationships that could be construed as a potential conflict of interest.
